# Confronting the challenges of the menopausal transition

**DOI:** 10.1186/s40695-015-0008-5

**Published:** 2015-10-05

**Authors:** Robert L. Reid, Bryden A. Magee

**Affiliations:** Division of Reproductive Endocrinology and Infertility, Queen’s University, Kingston, Ontario K7L 4 V1 Canada

**Keywords:** Menopause, Vasomotor symptoms, Menopausal hormone therapy

## Abstract

Canada’s Generation X is now entering the menopausal transition and pursuing effective therapy for bothersome vasomotor symptoms. They do so at a time when confusion about the safe and appropriate use of menopausal hormone therapy (MHT) has never been greater. Misplaced fears among women and their health care providers about MHT have, in many circumstances, led them to abandon this most effective therapy. This review discusses the physiology of the menopausal transition, the nature of symptoms related to withdrawal of ovarian estrogen production, and the potential benefits and risks of MHT. It is now clear that for most recently menopausal women the benefits of MHT outweigh the risks. The rationale for choosing different dosages, formulations, and regimens is reviewed.

## Introduction

Those involved in the care of menopausal women when the first Women’s Health Initiative (WHI) results were published in 2002 will remember that year as pivotal in the management of women entering the menopausal transition. The sensational way that negative results were fed to the media [1] triggered a cascade of events that created a fear of menopausal hormone therapy (MHT) leading women and their care providers to abandon the most effective treatment for menopausal vasomotor symptoms and substitute a variety of untested alternatives for which the risk-benefit profile was largely unknown.

**Fig. 1:**
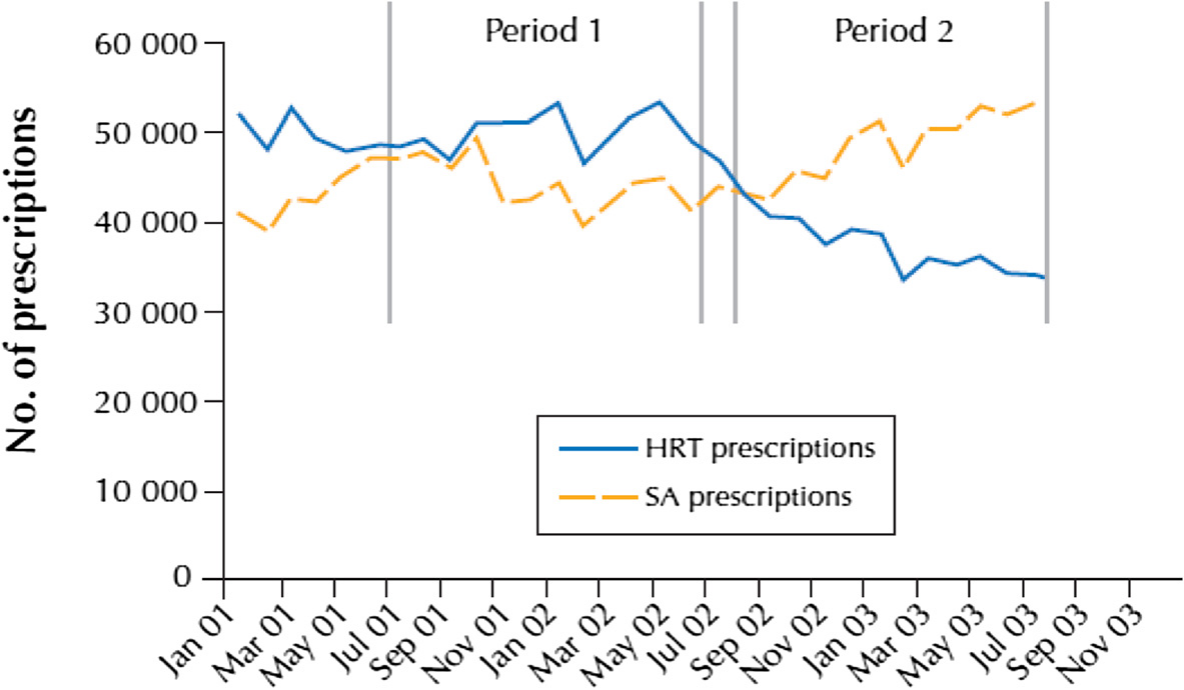
Prescriptions of MHT and Selective serotonin reuptake inhibitors in Canada showing a reciprocal trend after 2002. With permission from McIntyre RS, Konarski JZ, Grigoriadis S, Fan NC, Mancini DA, Fulton KA, Stewart DE, Kennedy SE. Hormone replacement therapy and antidepressant prescription patterns: a reciprocal relationship CMAJ 2005; 172 (1):57–59. Reproduced with permission of the Canadian Medical Association Journal

In Canada for example, a reciprocal relationship in prescribing practices for prescriptions of MHT and selective serotonin reuptake inhibitors (SSRIs) was seen, beginning in 2002. As shown in Fig. 1, the number of prescriptions written for antidepressants increased while those written for MHT quickly dropped off, suggesting that “antidepressants were being prescribed for symptoms (psychological, physical) previously controlled with the use of hormone replacement therapy” [2].

The absence of effective therapies for distressing vasomotor symptoms has contributed to a burgeoning market for complementary and alternative medicines (CAMs) and the unscrupulous marketing of purportedly “safer” hormone therapy in the form of individualized compounded bio-identical hormones [3]. There is, in fact, considerable evidence that many CAMs are adulterated and fail to contain the constituents as advertised. More so, most CAMs have yet to establish any scientific evidence of efficacy beyond a placebo effect [4]. Ultimately, there is no evidence that compounded bio-identical hormones are more effective or safer than regulated pharmaceuticals that have undergone rigorous clinical trials before reaching the market [5].

A significant shortcoming of the WHI was the inclusion of women well beyond the age of menopause. Women up to age 79 were eligible to participate and ultimately, 2/3^rds^ of recruited subjects were over age 60 years. A 2002 editorial warned of this flaw, suggesting that if the role of exercise for cardiovascular disease prevention had been tested in the same age group as the WHI, it is likely that the number of induced cardiac events would have led to the conclusion that exercise is bad for the heart [6]. A full decade after the original WHI report, a publication from several WHI lead investigators acknowledged that “the unfortunate effects of the WHI came not from problems with the design or the findings; rather, they were the result of generalizing findings from a well-conducted study to a subgroup that was not adequately represented”. In other words, the adverse consequences of MHT observed in older women were, in error, extrapolated to younger, newly menopausal women started on MHT. These authors further concluded: “With initiation of HT near menopause, the weight of evidence supports benefits over risks, with the potential to prevent or ameliorate downstream morbidity” [7].

Unfortunately, the damage from 10 years of adverse publicity about MHT has already been done. According to Vincent Convello from the Centre for Risk Communication at Columbia University “strong beliefs about risk, once formed, change very slowly and are extraordinarily persistent in the face of contrary evidence”. This seems particularly so for MHT. Despite the recent reanalyses of the WHI, which confirm that benefits outweigh risks for most newly menopausal women, fear and confusion continue to linger. This is no doubt exacerbated by the fact that rare adverse events remain a focus for media and for lawyers looking to add additional treatments to the 1–800 BAD-DRUG list.

Leading medical societies devoted to the care of menopausal women in 2012 published a consensus document to try to bring clarity to the benefits and risks of MHT and concluded that “there is no question that hormone therapy has an important role in managing symptoms for women during the menopausal transition and in early menopause” [8].

This reassurance however, is not enough because a generation of medical graduates have now been trained in an environment where use of MHT is frowned upon. This has resulted in limited or, in some cases, non-existent exposure to prescribing MHT or addressing side effects of treatment.

This review will summarize benefits and risks of MHT based on the best available evidence today and will provide practitioners with directions to assist them in prescribing MHT appropriately to women with disruptive symptoms during the midlife transition.

## Review

### Understanding the menopausal transition

The developing follicle is the primary source of estrogen in women during the reproductive years. In general, unless perturbed by pregnancy, extremes of body weight or other hormonal disorders, the menstrual cycle throughout the reproductive years tends to have a monthly periodicity. As the pool of oocytes diminishes, women in their 40’s often experience a decade of increasingly variable menstrual cycle length.

Between the ages of 40 and 45, the process of oocyte maturation accelerates, such that the follicular phase shortens to 7–8 days instead of the typical 14 days in younger women. An aberrant luteal phase elevation of estrogen (a so-called LOOP event [9]) leads to an early LH surge in the subsequent cycle and is considered one explanation for this abbreviated follicular phase. In other women, if the LOOP event does not result in ovulation, it may lead to delayed follicular development and prolongation of the subsequent cycle. Coupled with heavier and longer menses (often 8–10 days due to anatomic (adenomyosis or fibroids) or other factors (excessive prostacyclin or fibrinolytic activity), this menstrual cycle irregularity will result in many women seeking treatment for dysfunctional uterine bleeding.

Between the ages of 45 and 50, the remaining oocytes are those that have been most resistant to gonadotropin stimulation. Follicular development may halt temporarily until FSH levels rise sufficiently to force maturation. During intervals with arrested follicular development, estrogen levels fall and women experience the typical menopausal hot flashes and night sweats. Then, if and when follicular maturation resumes, estrogen levels rise and menopausal symptoms subside, only to be replaced by typical menstrual-cycle related symptoms such as breast tenderness, bloating and mood changes followed by often heavy and unpredictable menstruation.

The conventional term “perimenopause”, which was originally defined by the World Health Organization, encompasses the period of time from the onset of menstrual irregularity (usually in the mid 40’s) until one year after the final menstrual period when the postmenopausal phase begins [10]. More recently, the Stages of Reproductive Aging Workshop + 10 (STRAW +10), developed an updated staging system for ovarian aging in an attempt to standardize nomenclature. As such, the period of time from onset of menstrual irregularity to the final menstrual period is now defined as the “menopausal transition” [11].

It is hardly surprising that the perimenopause is a time of confusion and therapeutic frustration for women and their health care providers alike. The intermittent nature of clinical signs and symptoms of menopause leave uncertainty about whether the final menstrual period has occurred and with this variability, there may be confusion about the ongoing need for contraception, menstrual cycle regulation and/or hormone supplementation. Ultimately, most women can expect to exhaust their pool of oocytes by their mid-fifties with the average age of menopause being 51.5 years. Sustained amenorrhea with repeated elevation of FSH levels is diagnostic of menopause however therapy for distressing symptoms need not be delayed until the retrospective diagnosis of menopause has been proven.

Typical early menopausal symptoms include hot flashes and night sweats (vasomotor symptoms), and frequent night time wakenings with subsequent daytime fatigue or irritability [12]. Somatic aches and pains are the most frequent symptoms reported by women entering menopause [13, 14] but because most women assume that arthritic changes are a natural part of aging, vasomotor symptoms remain the most common reason for women to seek medical attention. In some women, mood changes may play a major role and clinical depression is a documented consequence in susceptible individuals [15]. Later on, prolonged estrogen deficiency plays a contributory role in the development of Genitourinary Syndrome of Menopause (GSM) – a condition marked by the gradual development of vaginal dryness and dyspareunia [16], increased risk for recurrent bladder or vaginal infections [17], and in some women, bladder overactivity.

Other effects of estrogen loss that lead to late clinical manifestations include accelerated bone loss (contributing to osteoporotic fractures) and certain adverse changes in cardiovascular risk factors such as lipids, obesity, diabetes, coronary artery calcium (CAC) accumulation and carotid intima-media thickness – all contributors to cardiovascular diseases [18]. Premature loss of endogenous estrogen has been shown to lead to earlier development of these menopause-associated conditions [19, 20].

### Benefits of MHT

Although estrogen therapy has been shown to diminish somatic aches and pains and to reduce new onset of joint symptoms in menopausal women [21, 22] this seems to have remained a well-guarded secret. MHT (estrogen alone, progestin alone, or combination therapy) is best known for the dramatic relief afforded from distressing vasomotor symptoms. Systemic estrogen supplementation, even in low doses, can achieve up to an 80 percent decrease in hot flashes and night sweats with the peak effect generally evident by 4 weeks of treatment [23, 24].

Vaginal estrogen dosages recommended for treatment of GSM result in minimal systemic absorption and are therefore not effective for systemic complaints such as joint pain or vasomotor symptoms. Higher doses of vaginal estrogen do achieve systemic levels sufficient to control vasomotor symptoms [25].

The use of MHT confers the additional benefit of protecting against the accelerated bone loss of menopause and should be considered first line treatment for bone protection in symptomatic women [26]. The benefits of estrogen for prevention of further bone loss and stabilization of bone mineral density are seen even when estrogen treatment is delayed for a number of years after menopause [27] but the accelerated bone loss of menopause resumes rapidly once estrogen is discontinued [28].

Systemic MHT sometimes, and vaginal estrogen therapy almost always, provide protection of urogenital tissues from the effects of diminished estrogen exposure after menopause. Vaginal epithelium maintains healthy rugation with a more normal stratified squamous cell layer, better blood flow, and improved secretion (transudate), all of which contribute to better lubrication, less dyspareunia, increased sensation, and greater sexual satisfaction. Intravaginal but not systemic estrogen appears to improve the symptom of urgency incontinence but remains only one component of a multifaceted approach to treatment of this condition [29].

The role of hormone therapy for prevention of coronary artery disease (CAD) has been the focus of considerable confusion and debate [30, 31]. Observational studies like the Nurse Health Study, which found that MHT users were half as likely to develop CAD, are prone to certain biases due to differences in patient characteristics and behaviours between the two groups. MHT users differed in other attributes from non-MHT users, in that they were more likely to adopt other health promotion strategies like regular exercise and a healthy diet [32]. The Women’s Health Initiative found that the use of MHT soon after menopause did delay the onset of coronary artery calcium (CAC) deposition [31, 33, 34] and that women with VMS had less CAC, which was attributed to prior MHT exposure [35]. In newly menopausal women MHT resulted in a small reduction in deaths due to CAD (1 fewer death per 1000 women years). However, MHT started in older women or those with pre-existing CAD increased the risk of adverse events [36–38]. This and observations from animal studies suggesting that healthy coronary arteries respond differently to estrogen than diseased vessels [39, 40] led to the “window of opportunity” theory for initiation of MHT while coronary arteries were still healthy [41].

We will probably never have the type of data needed to completely resolve this issue. It would be extremely difficult to recruit symptomatic newly menopausal women to a placebo controlled trial of sufficient duration to see outcome data for CAD. Depypere has calculated that it would be necessary to enrol over 185,000 women to detect a 10 % decrease in 10-year mortality [42].

MHT does appear to improve insulin sensitivity and reduce the incidence of new onset diabetes mellitus [43]. The WHI found 1 fewer case of new onset diabetes per 625 women years of use [44]. Recent evidence indicates that MHT has little impact on centripetal obesity, weight gain or blood pressure [45–47].

### Risks of MHT

#### Keeping perspective

As discussed above, the true risks of MHT have been inflated in the media, creating fear among many women and health care providers [48]. The persistence of this fear stems from a distorted perception of the apparent risks, and this distortion that has been demonstrated by a large Australian population survey [49]. Breast cancer was perceived as a major health risk by 27 % of women compared to only 11 % who cited heart disease as a concern. In contrast to perceived risk, actual female mortality figures show that these conditions account for 3 % and 41 % of mortality respectively.

## What are the risks of MHT in the perimenopausal or recently menopausal woman?

### Cardiovascular diseases

#### Venous Thromboembolism (VTE)

Venous thromboembolism includes deep vein thrombosis and pulmonary embolism. Advancing age and obesity are important contributors to VTE risk in women on MHT [50].

In the Women’s Health Initiative, women using MHT between the ages of 50–59 experienced 1 more case of VTE for every 1000 women per year [51]. This doubled for women 60–69 and tripled for women 70–79. Women with a body mass index (BMI) 25–30 kg/m^2^ had double the risk of women with BMI <25 kg/m^2^ and in those with BMI >30 kg/m^2^ the risk was tripled. Cigarette smoking does not appear to be a risk for VTE though it is clearly a risk factor for coronary artery disease and stroke [52].

Risk of VTE is greatest in the first year after initiation of MHT, just as is seen with combined hormonal contraception. Hence, the actual risk to women who have safely used MHT for several years is probably lower than short-term studies indicate.

Air travel has also been identified as an important contributor to VTE, likely due to cramped seating, immobility, hypoxia and dehydration. Recent research indicates that risk of symptomatic events is 1/600 for flights over 4 h and 1/500 for flights over 12 h in travellers over 50 years of age and that this risk may be doubled in women on MHT [53].

Observational data suggest that the risk of VTE can be reduced by using lower dose MHT or transdermal formulations [54]. Findings from the large prospective Million Women cohort study in the UK suggest that estrogen-only MHT or combined estrogen/progestin therapy (EPT) containing norgestrel/norethisterone progestins instead of medroxprogesterone actetate are also associated with lower VTE risk [55].

#### Stroke

Stroke remains a leading cause of morbidity and mortality in women, so any effect of MHT on stroke risk could be important. Results of past studies have been contradictory with some showing protection from ischemic stroke and others demonstrating small increases in risk. In the WHI study, hemorrhagic stroke risk was not influenced by MHT.

The attributable risk for ischemic stroke in the combined estrogen/progestin trial for all age groups was 0.9/1000 women years. For women ages 50–59 years the attributable risk of stroke was approximately 1/2500 women years of hormonal exposure [44, 56]. The attributable risk for all women enrolled in the estrogen alone arm of the trial was 1.1/1000 women years. Women aged 50–59 receiving estrogen alone showed no increase in the risk of stroke compared to placebo users [44, 57]. The impact of MHT on ischemic stroke seems to be related to hormone dosage and possibly also the route of delivery [58]. Although transdermal delivery has been suggested as a safer delivery route for MHT there is evidence that higher doses delivered transdermally may still increase the risk of stroke [59].

#### Coronary Artery Disease (CAD)

As per the discussion above, MHT may carry a small risk of a coronary event when started in women beyond age 60 or more than 10 years after menopause because coronary arteries may already be diseased by this time. The absolute increase in mortality in older MHT users in the WHI was small, at 1.6 per 1000 women years. Nevertheless it is clear that co-morbidities (diabetes, obesity, hypertension dyslipidemia, smoking, inactivity) should be addressed before initiating MHT in older women. The low absolute risk of adverse outcomes should not preclude a clinical trial of MHT for distressing VMS in women beyond age 60 or more than 10 years after menopause.

### Cancers

#### Endometrial cancer

Unopposed estrogen in women with a uterus may lead to endometrial neoplasia and in some cases this may become apparent years after MHT treatment has been stopped. The risk of progression from endometrial hyperplasia to cancer depends on the type of hyperplasia identified. When atypical features exist the risk of progression to adenocarcinoma may be as high as 30 % [60].

Progestin co-therapy of sufficient dosage and duration will generally protect against the risk of endometrial neoplasia associated with unopposed estrogens [61]. For many women, progestin co-therapy results in unwanted side effects (spotting, mood changes and bloating) so alternative approaches have been explored including systemic estrogen combined with a progestin releasing intrauterine system [62] or a combination of estrogen and a selective estrogen receptor modulator [63].

#### Breast cancer

Breast cancer remains the greatest fear of most women considering MHT. Much of the breast cancer anxiety can be attributed to the enormous success of breast cancer awareness campaigns and the accompanying “pink ribbon” merchandising of everything from Kentucky Fried Chicken and Campbell’s soup to jewelry and perfume [64]. The challenge for health care providers is to dispel distorted concepts about risk and to put true “absolute” or “attributable” risks into perspective [65, 66].

Table1 shows the actual risks of breast cancer for a hypothetical cohort of 1000 women over successive 10-year intervals compared to other (mostly cardiovascular) causes of mortality [67].

**Table 1:** Chances of the development of, and death from, breast cancer within the next 10 years for a cohort of 1000 women. With permission from Fletcher SW, Elmore JG. Clinical practice: Mammographic screening for breast cancer. NEJM 2003; 348(17):1672–1680 Reproduced with permission of Massachusetts Medical Society

Age	Cases of invasive breast cancer	Death from breast cancer (per 1000 women)	Death from any cause
40 yrs	15	2	21
50 yrs	28	5	55
60 yrs	37	7	126
70 yrs	43	9	309
80 yrs	35	11	670

The WHI reported a hazard ratio for breast cancer among women using combined estrogen/progestin therapy of 1.3 with an attributable risk of 8/10,000 users per year [68, 69]. In women randomized to estrogen-alone the hazard ratio for breast cancer was 0.77 indicating an attributable benefit of 7/10,000 fewer invasive breast cancers per year [70, 71]. While other data on the effects of estrogen on breast cancer are contradictory [72–74], the weight of evidence suggests that the breast cancer risk is lower with estrogen alone than with combined estrogen/progestin therapy and that cancers may not appear without longer term use [75].

The level of risk attributable to use of combined EPT for more than 5 years is equivalent to the risk associated with other biological determinants (early menarche, late menopause, first birth after age 30, failure to breast feed, postmenopausal obesity) or lifestyle choices (regular use of alcohol, failure to exercise etc.) [76, 77].

Based on the level of risk “attributable “ to MHT Collins has concluded that “When menopausal women present with distressing vasomotor symptoms, they can be reassured that short term (less than 5 years) use of either combined EPT or E alone will have little appreciable effect on their personal breast cancer risk. Longer term use increases risk to a level similar to risks that many women accept through lifestyles that expose them to daily alcohol ingestion, lack of regular exercise and postmenopausal obesity” [66].

For women who need MHT for control of distressing VMS and those at high risk for osteoporosis where other therapies may be poorly tolerated continued use beyond 5 years is appropriate after an individualized discussion of benefits and risks [78].

#### Colorectal cancer

Several studies have demonstrated a reduction in colorectal cancer incidence [79, 80] and mortality [81] in current users, but not former users of MHT. In the WHI, although the incidence of newly diagnosed colorectal cancers was lower in women on combined estrogen and progestin therapy, there was no survival difference due to more advanced stages of cancer in MHT users [82]. The weight of evidence does not support the use of MHT for colorectal cancer prevention.

#### Ovarian cancer

Several past studies have demonstrated a slight increase in the incidence of serous and endometrioid ovarian cancers and a decrease in mucinous ovarian cancers among women using hormone therapy [83, 84]. This risk disappeared within 2 years of cessation of MHT. The attributable risk was very small with an extra ovarian cancer occurring in 1/8,300 hormone users per year. The WHI did not find an increase in ovarian cancer incidence or mortality among MHT users [85].

#### Lung cancer

Reassuring data indicating that MHT has no effect on the incidence of lung cancer has come from several long-term observational studies [86]. A pooled analysis of 6 case control series involving close to 2000 lung cancer patients and a similar number of controls reported by the International Lung Cancer Consortium even suggested that MHT might reduce the risk of lung cancer by 25-30 % [87]. Nevertheless, combined estrogen/progestin therapy has been suggested as a modifying factor in the progression of lung cancer [88]. In the WHI, although MHT did not significantly alter the incidence of lung cancer, mortality from lung cancer was increased in women using combined estrogen/progestin therapy [85].

### Gall bladder disease

Published data have consistently shown that gallbladder disease (gallstones and cholecystectomy rates) are increased in current and former users of MHT (estrogen alone more than combined estrogen/ progestin, and, oral MHT more than transdermal MHT) [89, 90]. In the WHI, there was one additional case of gallbladder disease per 200 women years of MHT use [44]. Estimates suggest that transdermal MHT would avoid 1 cholecystectomy for every 600 women years of use [91].

## Addressing the needs of the perimenopausal and menopausal woman

### The symptomatic perimenopausal woman

Therapy for intermittent menopausal symptoms (hot flashes and night sweats) of the perimenopause need not be delayed until menopause is confirmed. Rather it is appropriate to begin treatment as soon as distressing symptoms appear. Standard MHT may offer a dosage that is too low to override the intermittent ovarian activity that characterizes the perimenopause. In this case a low dose combined hormonal contraceptive may be ideal for low risk non-smokers since it provides menstrual cycle regulation, suppression of vasomotor symptoms, and contraception. For smokers or individuals at higher risk for cardiovascular complications (obesity, immobility or diabetes) an alternative would be to combine a progestin releasing intrauterine system with systemic transdermal estrogen. The device will suppress bleeding and afford contraception while the transdermal estrogen alleviates vasomotor symptoms.

### The symptomatic menopausal woman

For women who present after menopause is clearly established it is first important to elucidate the nature and severity of symptoms, risk factors for CVD, osteoporosis and cancer, and the extent of any co-morbidities. Ideally co-morbidities like diabetes, hypertension or dyslipidemia should be addressed before MHT is commenced. Before MHT is started a physical examination is generally wise to evaluate for hypertension or any pre-existing breast or pelvic abnormalities. An endometrial biopsy or ultrasound to assess the endometrium should be considered if any abnormal uterine bleeding has occurred in the preceding months.

After a review of anticipated benefits and possible risks, a joint decision between patient and practitioner should be reached about the initial dosage, route of delivery, type of estrogen and progestin and regimen. Estrogen, either by itself or with progestins, is the most consistently effective therapy for hot flashes and night sweats.

Low-dose estrogen (doses of 0.3 mg of conjugated equine estrogen, 0.5 mg of oral micronized estradiol, 25 ug of transdermal estradiol, or 2.5 mg of ethinyl estradiol) has been shown to be effective for many women, although some women require a higher dose for relief of hot flashes [92, 93]. No convincing clinical evidence suggests that one product is safer or more efficacious than the other. Initial relief from hot flashes is generally rapid (within 1 week) but may take longer when treatment is initiated with these very low doses [24].

In the absence of specific risk factors, route of delivery can often be decided by the woman who will know best how she can maintain therapy. Observational studies suggest that transdermal delivery may be associated with a reduced risk of VTE and possibly stroke [94]. Therefore when specific risk factors for these conditions exist (smoking, obesity, immobility, or a known personal/family history of thrombophilia) a transdermal approach should be recommended.

Which progestin to use in women with a uterus has been the subject of considerable debate. Medroxyprogesterone acetate (MPA) has been widely employed in MHT throughout North America for several decades. It was chosen for the WHI investigation because of its popularity and widespread use at the time. Since then questions have surfaced about the relative safety of different progestins with some arguing for a switch to natural progesterone [92] and others arguing that experimental data on MPA have not translated to clinical outcomes and that continued short term use of MPA is safe [95]. Used cyclically the progestin dosage is MPA 5 mg or progesterone 200 mg for 10–12 days per month. With very low doses of estrogen, especially if progestin causes side effects, progestin treatment may be administered less frequently (every two or three months). For continuous combined therapy the usual progestin dosage is MPA is 2.5 mg or progesterone is 100 mg daily. Other specific combined formulations offer a fixed dose of estrogen and progestin in a single tablet.

Traditionally, MHT employed a regimen that allowed a one week hormone-free interval per month during which time a small amount of scheduled bleeding might occur. At these very low doses there is often minimal endometrial growth and little, if any, withdrawal bleeding. On the other hand, the hormone free week is often punctuated by return of distressing vasomotor symptoms. This has led to a variety of continuous regimens employing a daily combination of estrogen and progestin in women with a uterus or estrogen alone for women after hysterectomy. Personal tolerance to spotting or preferences for scheduled cyclic bleeding may help determine whether a continuous combined MHT regimen would be preferred to a cyclic regimen with monthly withdrawal bleeding.

Counselling should include what initial side effects to anticipate (breast tenderness, spotting, discharge etc.) and when a follow-up evaluation is warranted (unscheduled bleeding after 6 months). Generally, since the peak benefit for vasomotor symptoms occurs at 1 month after initiation, a follow-up appointment in two months works well for a review. Remember to enquire about whether the systemic therapy is adequately correcting any vaginal dryness and dyspareunia – if not, add a prescription for vaginal estrogen. If symptom control is good, and the woman is satisfied, a one-year prescription and annual review is appropriate.

Regular monitoring of bone mineral density is not required while on MHT as long as there are no other significant risk factors for osteoporosis [19]. MHT may be continued for as long as the distressing symptoms persist for which the treatment was started. A brief (eg. 2 week) break from MHT will allow women to rapidly discern whether continued treatment is necessary for relief of symptoms. Among women who stopped MHT after the publication of the WHI, 25 % resumed therapy, presumably due to severity of symptoms [93, 96]. Periodic review of the need for treatment and an individualized discussion of benefits and risks is appropriate when the prescription is renewed. Often women will realize when therapy is no longer needed for control of vasomotor symptoms and this would be an appropriate time to discontinue systemic therapy. When systemic treatment is stopped it is important for the health care provider to counsel about the possible development of Genitourinary Syndrome of Menopause and therapeutic options available to prevent this. As well, in anticipation of accelerated bone loss in the next few years, a baseline BMD with follow-up in 1.5 - 2 years is appropriate.

## Conclusions

The perimenopause and menopause are periods of life that can disrupt the quality of life for many women, so an understanding of the pathophysiology and the potential consequences of waning ovarian estrogen production can inform accurate counselling and management. Most women considering MHT at this time of life can be informed that risks are few and that in fact, the benefits extend well beyond the control of vasomotor symptoms that precipitated the visit. Regular follow-up with attention to co-morbidities and periodic review of individual benefits and risks will allow for optimal duration of treatment.

## Abbreviations

CAC: Coronary artery calcification
CAD: Coronary artery disease
CAM: Complementary and alternative medicine
CVD: Cardiovascular disease
EPT: Estrogen and progestin therapy
GSM: Genitourinary syndrome of menopause
MHT: Menopausal hormone therapy
MPA: Medroxyprogesterone acetate
SSRI: Selective serotonin reuptake inhibitors
VSM: Vasomotor symptoms
WHI: Women’s health initiative
